# The Systolic Pulmonary Arterial Pressure Liaises Impaired Cardiac Autonomic Control to Pro-inflammatory Status in Systemic Sclerosis Patients

**DOI:** 10.3389/fcvm.2022.899290

**Published:** 2022-07-01

**Authors:** Gabriel D. Rodrigues, Marco Vicenzi, Chiara Bellocchi, Lorenzo Beretta, Angelica Carandina, Eleonora Tobaldini, Stefano Carugo, Nicola Montano

**Affiliations:** ^1^Department of Clinical Sciences and Community Health, University of Milan, Milan, Italy; ^2^Post Graduation Program in Cardiovascular Sciences, Federal Fluminense University, Niterói, Brazil; ^3^Cardiovascular Disease Unit, Fondazione IRCCS Ca’ Granda Ospedale Maggiore Policlinico, Milan, Italy; ^4^Dyspnea Lab, Department of Clinical Sciences and Community Health, University of Milan, Milan, Italy; ^5^Department of Internal Medicine, Fondazione IRCCS Ca’ Granda Ospedale Maggiore Policlinico, Milan, Italy

**Keywords:** heart rate variability, scleroderma, symbolic analysis, inflammatory reflex, autoimmune diseases

## Abstract

The current study was undertaken to test the hypothesis that systemic sclerosis (SSc) patients with higher systolic pulmonary arterial pressures (PAPs) present a blunted cardiac autonomic modulation and a pro-inflammatory profile. Thirty-nine SSc patients were enrolled (mean age 57 ± 11 years). ECG and respiration were recorded in the supine (SUP) position and during the active standing (ORT). Heart rate variability (HRV) analysis was performed on samples of 300 beats. The symbolic analysis identified three patterns, 0V%, (sympathetic) and 2UV% and 2LV%, (vagal). The %ΔORT was calculated from the differences between HRV in ORT and SUP, normalized (%) by the HRV values at rest. The PAPs was obtained non-invasively through echocardiography. For the inter-group analysis, participants were allocated in groups with higher (+PAPs ≥ median) and lower PAPs (–PAPs < median) values. At rest, the cardiac sympathetic modulation (represented by 0V%) was positively correlated with PAPs, while parasympathetic modulation (represented by 2LV%) was negatively correlated with PAPs. The dynamic response to ORT (represented by Δ0V% and Δ2LV%), sympathetic and parasympathetic were negatively and positively correlated with PAPs, respectively. The +PAPs group presented a higher inflammatory status and a blunted cardiac autonomic response to ORT (↓Δ0V% and ↑Δ2LV%) compared to the –PAPs group. These findings suggest an interplay among cardiac autonomic control, inflammatory status, and cardiopulmonary mechanics that should be considered for the assessment, monitoring, and treatment of SSc patients.

## Introduction

Systemic Sclerosis (SSc) is an autoimmune disease characterized by vascular damage, autoantibodies production, and fibrosis of the skin and internal organs. SSc patients are classified into diffuse cutaneous SSc (dcSSc) or limited cutaneous SSc (lcSSc) subsets based on the extent of their skin fibrosis (1–4). The heart and lungs are frequently affected in SSc with several manifestations including conduction abnormalities, myocarditis, coronary artery disease, valvular heart disease, pericardial manifestations, associated pulmonary arterial hypertension (APAH), myocardial and pulmonary fibrosis (1–6). Due to unspecific symptoms (typically dyspnea and fatigue), cardiopulmonary impairments are often diagnosed late leading to a poor prognosis. Clinically evident cardiac involvement is associated with up to 70% mortality at 5 years (1, 7, 8). In this turn, APAH is also a relevant cause of death in SSc, despite an optimized treatment (2, 5). The non-invasive monitoring of the integrity of the pulmonary circulation through regular Doppler echocardiography and/or the functional assessment through the exercise cardiopulmonary testing should be considered in early APAH screening (9, 10).

Autonomic dysfunction was identified as an early marker of SSc progression, helping to identify cardiac involvement (11, 12). Heart rate variability (HRV) has been reported as a powerful non-invasive tool to access heart sympathetic and vagal modulation in SSc disease (11, 13). HRV predicted severe myocardial damage that is linked to ventricular arrhythmias in patients with SSc (12). Recently, HRV linear and non-linear analysis revealed that cardiac autonomic impairment follows the fibro-vascular progression in SSc. Indeed, the dcSSc subset shows a more pronounced shifted sympatho-vagal balance (i.e., sympathetic predominance and vagal withdrawal) if compared to the lcSSc in which there is a less extent of fibrosis (3). Moreover, SSc at a pre-clinical stage (14) shows an autonomic profile comparable to healthy controls (13).

In idiopathic PAH, high PAPs values were negatively correlated with vagal-mediated HRV linear indexes in time and frequency domains, and cardiac vagal modulation was lower in idiopathic PAH compared to age-matched healthy controls (15). In the same way, the augmented sympathetic discharge to the periphery was positively correlated with PAPs, highlighting that the sympathetic nervous system plays a role in the pulmonary vascular disease pathophysiology (16).

Whether sympatho-vagal balance is modulated in patients with SSc is not well described and, to our knowledge, no studies investigated the association between PAPs and cardiac autonomic modulation (i.e., HRV) in SSc patients without features of APAH. Since cardiac autonomic control is impaired in both SSc (13) and idiopathic PAH (15), we could hypothesize an association between PAPs and HRV indexes in SSc. Moreover, inflammation might be a relevant bridge between autoimmune diseases and APAH, as a possible key mechanism underlying APAH development in SSc (17, 18).

Thus, the current study was undertaken to test the hypothesis that high pulmonary arterial pressure liaises impaired cardiac autonomic function with low-grade inflammation in SSc patients, even without clinically evident APAH.

## Materials and Methods

### Sample

This observational study included 39 SSc patients without a diagnosis of APAH. SSc patients were classified into dcSSc or lcSSc subsets based on the extent of their skin fibrosis (3). Patients with a definite SSc but without skin fibrosis yet with puffy fingers were categorized in the lcSSc group. The protocol was approved by the local Ethics Committee (Comitato Etico Milano Area 2), and all participants signed informed written consent before participation in the study.

### Clinical Features

Clinical and laboratory parameters were extracted from medical records for the determination of total lung capacity (TLC), forced vital capacity (FVC), forced expiratory volume in 1 s (FEV_1_), diffusing capacity for carbon monoxide (DLCO), left ventricular ejection fraction (LVEF) computed using the modified Simpson’s formula, tricuspid annular plane systolic excursion (TAPSE, normal value when > 16 mm), erythrocyte sedimentation rate (ESR, normal value from 1 to 20 mm/h), SSc-associated autoantibodies (ACA and aSCL70 positivity), C-reactive protein (CRP, normal value < 0.5 mg/dL), creatinine, glucose levels, and the presence of hypergammaglobulinemia.

According to the current recommendation, right atrial pressure (RAP) was estimated from the spontaneous changes of the inferior vena cava dimension during the expiratory-inspiratory phase, and set in different levels (5, 10, or 15 mmHg) according to the current guideline for the echocardiographic assessment of the right heart in adults (19). Doppler sampling of tricuspid regurgitation velocity was used to derive the right atrial-ventricular gradient through the simplified Bernoulli’s equation. Non-invasive PAPs estimate was obtained through echocardiography adding the RAP to the right atrial-ventricular gradient (19). The APAH was clinically ruled out according to the following exclusion criteria (9, 10): (1) DLCO < 60%; and (2) NYHA functional class ≥ 2.

### Cardiac Autonomic Modulation Assessment

All the participants underwent the recording of ECG and respiration (thoracic belt record) by an *ad hoc* telemetric system device (BT16 Plus, Marazza Spa, Monza, ITA). The signals were recorded at rest (supine position for 5 min) and during active standing (orthostatic position for 5 min) with spontaneous breathing. The absence of a stable sinus rhythm on ECG and ongoing therapy with beta-blocker drugs were considered exclusion criteria for this study.

The HRV was evaluated through specific software (Heart Scope II, AMPS, ITA) on short samples of 300 beats at supine (SUP) and orthostatic (ORT) positions. To assess the autonomic dynamic response to ORT, we calculated the ΔORT% (HRV in SUP position–HRV in ORT position/HRV in SUP position).

Non-linear dynamics of HRV were evaluated by symbolic analysis. The R-R dynamics were classified into three patterns families: (a) patterns with no variation (0V; all three symbols were equal); (b) patterns with one variation (1V; two consequent symbols were equal and the remaining symbol was different); and (c) patterns with two liked (2LV) and unlike (2UV) variations (e.g., all symbols were different from the previous one). The percentage of the patterns with no variation (0V) and with two variations (2UV or 2LV) were calculated as a predominance of sympathetic and parasympathetic cardiac autonomic modulation, respectively (13). However, the pattern of 1V was not associated with any autonomic tests used to validate the method (20).

### Statistical Analysis

The Shapiro–Wilk test was used to evaluate the normality of the samples. The Spearman’s correlation was used to test the association between PAPs and HRV indexes. Mann–Whitney *U* test was used for the inter-group analysis. The analysis of covariance (ANCOVA) was employed, using age as a co-variable to confirm the results from the inter-group analysis. For descriptive analysis, the median and the interquartile range (IQR 25–75%) were calculated for PAPs values. For the other variables, means and standard deviation were used. *P*-value < 0.05 was considered statistically significant. The software used was SPSS Statistics version 21.0 (IBM Corp., Armonk, NY, United States) and GraphPad Prism version 8.0 (GraphPad Software Inc., San Diego, CA, United States).

## Results

Our cohort was mainly composed of females and lcSSc subjects (77%) with a mean age of 57 ± 11 yrs. Overall clinical features are listed in Table 1. Mean disease duration was >than 3 years. All participants had a DLCO > 60% and belonged to the NYHA functional class I, thus excluding suspicion of APAH diagnosis. After patients’ allocation into two subgroups based on PAPs values above or below the median (median 30, IQR 27–34 mmHg; the +PAPs and –PAPs groups, respectively), significantly higher CRP levels were found in the +PAPs (1.01 ± 1.04 mg/dL) compared to the –PAPs group (0.40 ± 0.25 mg/dL; *p* = 0.02). No other clinical differences were found between +PAPs and –PAPs groups, except for higher age in the +PAPs. To note, these results were confirmed also after ANCOVA, which included age and SSc subset as co-variables.

**TABLE 1:** Participants’ characteristics and clinical features.

	All	-PAPs	+PAPs	*p*-value
**Personal characteristics**				
*N*	39	19	20	–
Sex (M/F)	9/30	4/15	6/14	–
Age (years)	57 ± 11	56 ± 10	64 ± 11	0.03*
SSc subsets (dcSSc/lcSSc)	6/33	3/16	3/17	–
Disease duration (years)	11 ± 8	10 ± 8	13 ± 9	0.17
Controlled arterial hypertension (*n*)	4	2	2	–
**Antibodies**				
Anti-Scl-70+ (*n*)	11	7	4	–
ACA+ (*n*)	17	7	10	–
ANA+ (*n*)	11	7	4	–
**Spirometry**				
TLC (L)	5.33 ± 1.30	5.17 ± 1.44	5.43 ± 1.24	0.72
FVC (L)	3.20 ± 1.00	3.43 ± 1.03	3.03 ± 0.96	0.30
FEV_1_ (L)	2.48 ± 0.92	2.72 ± 1.15	2.30 ± 0.67	0.08
DLCO SB, ml/mmHg/min	24.16 ± 35.35	16.52 ± 10.27	30.55 ± 46.92	0.58
DLCO SB, %	80 ± 17%	83 ± 12%	77 ± 20%	
DLCO SB/VA, ml/mmHg/min/L	7.65 ± 15.35	4.44 ± 1.59	9.52 ± 19.34	0.46
DLCO SB/VA, %	90 ± 18%	92 ± 10%	90 ± 22%	
**Echocardiography**				
LVEF (%)	61 ± 12	59 ± 16	63 ± 6	0.60
TAPSE (mm)	23 ± 5	24 ± 5	22 ± 5	0.39
PAPs (mmHg)	31 ± 8	25 ± 4	37 ± 6	<0.001*
**Biochemical**				
CRP (mg/dL)	0.73 ± 1.01	0.40 ± 0.25	1.01 ± 1.04	0.02*
ESR (mm/hour)	22 ± 18	20 ± 12	24 ± 12	0.84
γ-globulin (g/dL)	16.94 ± 7.07	16.70 ± 4.09	17.31 ± 10.78	0.74
Creatinine (mg/dL)	0.84 ± 0.21	0.76 ± 0.08	0.93 ± 0.30	0.26
Glucose (mg/dL)	87 ± 9	89 ± 10	84 ± 5	0.28

SSc, systemic sclerosis; lcSSc, limited cutaneous SSc; dcSSc, diffuse cutaneous SSc; Anti-Scl-70+, anti-topoisomerase I; ACA+, anti-centromere antibodies positive; TLC, total lung capacity; FVC, forced vital capacity; FEV1, forced expiratory volume in 1 s; DLCO SB, diffusing capacity for carbon monoxide; DLCO SB/VA, diffusing capacity corrected for alveolar volume; LVEF, left ventricular ejection fraction; TAPSE, tricuspid annular plane systolic excursion; PAPs, mean pulmonary artery pressure; CRP, C reactive protein; ESR, erythrocyte sedimentation rate; PAPs-, PAPs group of PAPs < median; PAPs+, group of PAPs ≥ median; *p < 0.05 from Mann–Whitney U test (–PAPs vs. +PAPs).

**FIGURE 1:**
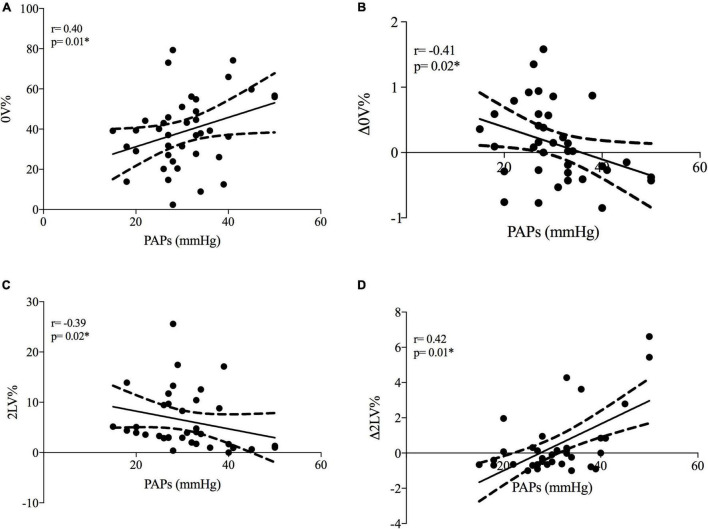
Correlations between systolic pulmonary artery pressure and cardiac autonomic modulation in SSc patients. PAPs, systolic pulmonary artery pressure; Δ0V%, [(0V% in supine position-0V% in orthostatic position)/0V% in supine position]. 0V% and 2LV% are non-linear heart rate variability markers of sympathetic and vagal modulation. Plots **(A,B)** represent cardiac sympathetic modulation at rest and sympathetic dynamic response to ORT, respectively. Plots **(C,D)** represent cardiac vagal modulation at rest and vagal dynamic response to ORT, respectively. *p < 0.05 from Spearman’s correlation.

At rest, PAPs positively correlated with cardiac sympathetic modulation (represented by 0V%) and negatively correlated with vagal modulation (represented by 2LV%) (see Figures 1A,C).

**FIGURE 2:**
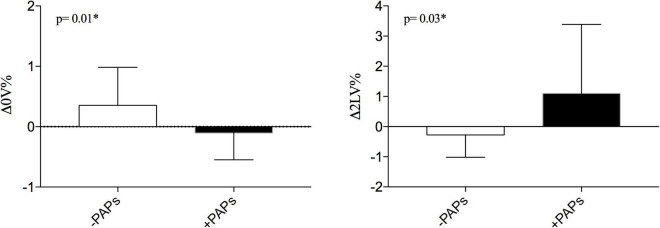
Comparisons of dynamic cardiac response to active standing test between –PAPs and +PAPs groups between. PAPs, mean pulmonary artery pressure; PAPs-, PAPs group of PAPs < median; PAPs+, group of PAPs ≥ median; Δ0V%, [(0V% in supine position-0V% in orthostatic position)/0V% in supine position]. 0V% and 2LV% are non-linear heart rate variability markers of sympathetic and vagal modulation. *p < 0.05 from Mann–Whitney *U* test.

When the autonomic dynamic response to ORT was assessed, the Δ0V% was negatively and Δ2LV% positively correlated with PAPs values (see Figures 1B,D). Other clinical features were not associated with HRV. As shown in Figure 2 the +PAPs group presented a blunted autonomic response to orthostatic stress (lower Δ0V% and higher Δ2LV%) compared to the –PAPs group, while at rest HRV indexes did not show differences between –PAPs and +PAPs groups.

## Discussion

This study showed that in SSc patients without a diagnosis of APAH: (1) at rest, PAPs values are positively correlated with sympathetic- and negatively correlated with vagal-mediated indexes of symbolic HRV analysis; (2) higher PAPs values are correlated to a blunted cardiac autonomic responses at active standing test (i.e., reduced Δ0V% and increased Δ2LV% and 2LV; (3) the +PAPs group presented higher CRP values (attributable to a low-grade inflammation) and a blunted cardiac autonomic modulation compared to the –PAPs group. Overall, our findings suggest that a cardiac autonomic assessment through a non-invasive tool such as HRV and active standing test, may unmask a preclinical derangement of cardiopulmonary circulation.

### Autonomic Nervous System and Cardiopulmonary Mechanics in Systemic Sclerosis and Associated Pulmonary Arterial Hypertension

From a previous study in idiopathic pulmonary arterial hypertension, vagal-mediated HRV was negatively associated with high values of invasively assessed PAPs (15). Indeed, a cardiac vagal withdrawal seems to be a consequence of an increased sympathetic drive, suggesting that cardiac vagal modulation is suppressed by sympathetic overactivity (13, 15). In line with these observations, our results showed that the sympathetic- and vagal-mediated indexes of HRV symbolic analysis were correlated with PAPs. Moreover, a previous study described a cardiac autonomic dysfunction in SSc patients with APAH (21). However, further studies with others HRV approaches and a more representative sample size are needed to confirm this finding.

As well known, the hemodynamic determinants of pulmonary pressure are represented by: (1) the cardiac output (CO) that determines the amount of blood flow; (2) the left ventricular end-diastolic pressure (LVEDP) that reflects the impact of the post-capillary component; and (3) the arterial pulmonary circulation, that reflects the pre-capillary component (22). In this complex hemodynamic scenario, the increased LVEDP, as well as the decreased CO, and impaired baroreflex control seem to be all factors of sympathetic overactivity in left heart disease (LHD) (23). Similarly, in severe PAH, a right ventricular (RV) failure, due to the pulmonary vascular remodeling, can reduce the systemic CO, provoking a compensatory sympathetic response (24). Moreover, an impaired baroreflex (16) and chemoreflex (25) sensitivities are associated with disease severity in PAH. Otherwise, it was suggested that the peripheral chemoreflex was not a direct mediator of cardiac baroreflex dysfunction in PAH (26). Thus, the sympathetic overactivity in LHD and PAH could be explained, in part, because of a compensatory response from an augmented neurohumoral activation to compensate harmed cardiac output mechanics (27).

From our data, an augmented sympathetic heart modulation was positively correlated with pulmonary arterial pressure values irrespective of the cause of PAPs increasing, suggesting that cardiac sympathetic activity could be a reactivity to the blunted response of the right heart and the pulmonary circulation (28, 29). The initially altered RV after-load induces a compensatory reaction of RV itself in order to reduce hemodynamic stress and preserve contractility (e.g., normal values of TAPSE). These compensatory adaptations were linked with enhanced sympathetic activity, which in PAH is actively involved in the pulmonary vascular disease progression (28, 29). Thus, the interplay between sympathetic overactivity and augmented PAPs might be an early marker of RV dysfunction in SSc patients, which deserves further studies.

### The Interplay Among Cardiopulmonary Hemodynamic, Autonomic Control, and Inflammation

Our findings are consistent with the concept of the physiological “reserve” of the autonomic nervous system (30, 31), highlighting that the high variation (Δ) is related to better cardiovascular adjustments during autonomic stress tests and orthostatic tolerance (30). From our results, while the +PAPs group seems to present a lower autonomic reserve, the –PAPs group is closer to a normal physiological response to active standing (e.g., increased sympathetic response with a vagal withdrawal).

From the inter-group analysis, the +PAPs group shows higher CRP values compared to the –PAPs group (Table 1), with a biological meaning of low-grade inflammation (defined as CRP < 1.0 mg/dL) (32, 33). The low-grade chronic inflammation plays an essential role in the extracellular matrix deposition process leading to cardiopulmonary fibrosis, which represents a major cause of mortality in SSc (34–36). Moreover, in chronic cardiovascular diseases, low-grade inflammation is a marker of vascular disease leading to progression of atherosclerosis and driving ischemic events (32, 33, 37). It is conceivable that a slightly altered inflammatory status induces systemic and pulmonary vascular dysfunction and remodeling with consequent impaired cardiac autonomic modulation. These hypotheses are worthy to be further investigated in SSc.

### Clinical Applications and Future Directions

Our data showed that inflammation is related to HRV and high PAPs. Besides the standard treatment of SSc, which aims to control the inflammatory status and disease progression, other type of treatment should be considered. For future directions pharmacological and non-pharmacological approaches that stimulate the vagus nerve activity could be employed to reduce inflammation *via* the anti-inflammatory cholinergic reflex (38, 39).

### Limitations

Our study has some limitations. We estimated PAPs by echocardiogram but an invasive measurement of PAPs may assist a better recognition of physiological mechanisms. Moreover, the absence of other parameters of RV structure and function is limiting a comprehensive description of right heart remodeling/overload. The small number of dcSSc enrolled precluded further analyses across different SSc subsets. However, the prevalence of cardiovascular disorders (e.g., arterial hypertension, diastolic dysfunction, altered PAPs values) is not different between lcSSc and dcSSc sub-types (40). Thus, once APAH was excluded, we can suppose that the inter-group analysis is not affected by other bias of selection. Also, we did not evaluate additional markers of inflammation alternative to CRP (such as IL-6, TNF). Further studies with a larger sample size are needed to confirm our observations.

## Conclusion

Augmented PAPs plays a role in cardiac autonomic control in a cohort of SSc without APAH. High PAPs values were associated with a cardiac sympathetic predominance and a pro-inflammatory status in SSc. These findings encourage the inclusion of HRV analysis (linear and non-linear methods) and active standing test in the screening of PAH in SSc. Further studies might be conducted to investigate the putative mechanisms underlying the interplay of cardiopulmonary, inflammatory, and autonomic impairments in SSc, such as inflammatory reflex, baroreflex, and chemoreflex control.

## Data Availability Statement

The raw data supporting the conclusions of this article will be made available by the authors, without undue reservation.

## Ethics Statement

The studies involving human participants were reviewed and approved by Comitato Etico Milano Area 2. The patients/participants provided their written informed consent to participate in this study.

## Author Contributions

GR, MV, and CB: substantial contributions to the conception or design of the work and drafting the work. GR, MV, AC, and CB: acquisition and analysis. GR, MV, LB, ET, and CB: interpretation of data for the work. GR, MV, CB, LB, AC, ET, SC, and NM: revising it critically for important intellectual content. GR, MV, CB, LB, AC, ET, SC, and NM: final approval of the version to be published, agreement to be accountable for all aspects of the work in ensuring that questions related to the accuracy, and integrity of any part of the work are appropriately investigated and resolved. SC, ET, and NM: supervision. SC and NM: funding. All authors have read and agreed to the published version of the manuscript.

## Conflict of Interest

The authors declare that the research was conducted in the absence of any commercial or financial relationships that could be construed as a potential conflict of interest.

## Publisher’s Note

All claims expressed in this article are solely those of the authors and do not necessarily represent those of their affiliated organizations, or those of the publisher, the editors and the reviewers. Any product that may be evaluated in this article, or claim that may be made by its manufacturer, is not guaranteed or endorsed by the publisher.
